# FSH Therapy in Male Factor Infertility: Evidence and Factors Which Might Predict the Response

**DOI:** 10.3390/life14080969

**Published:** 2024-07-31

**Authors:** Giuseppe Grande, Andrea Graziani, Raffaele Scafa, Andrea Garolla, Daniele Santi, Alberto Ferlin

**Affiliations:** 1 Unit of Andrology and Reproductive Medicine, Department of Medicine, University of Padua, 35128 Padua, Italy; agraziani1995@gmail.com (A.G.); raffaele.scafa@studenti.unipd.it (R.S.); andrea.garolla@unipd.it (A.G.); alberto.ferlin@unipd.it (A.F.); 2 Department of Biomedical, Metabolic and Neural Sciences, University of Modena and Reggio Emilia, 41125 Modena, Italy; daniele.santi@unimore.it; 3 Unit of Endocrinology, Department of Medical Specialties, Azienda Ospedaliero-Universitaria of Modena, 41126 Modena, Italy; 4 Unit of Andrology and Sexual Medicine of the Unit of Endocrinology, Department of Medical Specialties, Azienda Ospedaliero-Universitaria of Modena, 41126 Modena, Italy

**Keywords:** male factor infertility, infertility, FSH, FSHB, FSHR, semen analysis

## Abstract

Follicle-stimulating hormone (FSH) administration is applied in the management of subjects affected by hypogonadotropic hypogonadism. Whilst this application is widely recognized and established alone or in combination with human chorionic gonadotropin (hCG), a similar strategy is empirically advocated in idiopathic male factor infertility (MFI). In this setting, FSH therapy has been used to increase sperm quantity, quality, and pregnancy rate when FSH plasma concentrations are below 8 IU/L and when the seminal tract is not obstructed. In the literature, several studies suggested that giving FSH to patients with idiopathic MFI increases sperm count and motility, raising the overall pregnancy rate. However, this efficacy seems to be limited, and about 10–18 men should be treated to achieve one pregnancy. Thus, several papers suggest the need to move from a replacement approach to an overstimulating approach in the management of FSH therapy in idiopathic MFI. To this aim, it is imperative to determine some pharmacologic markers of FSH efficacy. Furthermore, it should be useful in clinical practice to distinguish, before starting the treatment, among patients who might respond or not to FSH treatment. Indeed, previous studies suggest that infertile men who have normal levels of gonadotropins in plasma might not respond to FSH treatment and about 50% of patients might be defined as “non-responders”. For these reasons, identifying predictive markers of FSH action in spermatogenesis and clinical markers of response to FSH treatment is a fascinating area of study that might lead to new developments with the aim of achieving personalization of the treatment of male infertility. From this perspective, seminal parameters (i.e., spermatid count), testicular cytology, genetic assessment, and miRNA or protein markers in the future might be used to create a tailored FSH therapy plan. The personalization of FSH treatment is mandatory to minimize side effects, to avoid lost time with ineffective treatments, and to improve the efficacy, predicting the most efficient dose and the duration of the treatment. This narrative review’s objective is to discuss the role of the different putative factors which have been proposed to predict the response to FSH treatment in idiopathic infertile men.

## 1. Background

### 1.1. Male Factor Infertility

The World Health Organization (WHO) defines an infertile couple as being unable to conceive following a year or more of regular, unprotected sexual activity [1]. It is a common condition, and in 10–15% of couples globally, male factor infertility (MFI) is present, either by itself or in conjunction with a female component, in around half of the total instances [2,3].

Despite all of the advances in our understanding of MFI, about 20–50% of cases remain without a definite cause [4], being defined as “idiopathic” MFI. Indeed, this term ought to be used only in situations where, following a thorough, exhaustive, and precise diagnostic procedure, no cause can be found [3,5]. The high proportion of idiopathic MFI suggests that the mechanisms regulating spermatogenesis and sperm function are largely unknown [4]. Indeed, idiopathic MFI is the condition in which the diagnostic process does not allow for the identification of the cause.

Several hormonal approaches have been developed to improve sperm concentration in patients with idiopathic oligozoospermia, such as the use of FSH and the off-label use of clomiphene and letrozole/anastrozole.

### 1.2. FSH Therapy: Evidence and Current Use

The pituitary gland secretes follicle-stimulating hormone (FSH), a dimeric glycoprotein that targets both male and female gonadal cells. The molecule bears structural similarities to luteinizing hormone (LH), which works in tandem with FSH to regulate reproduction by influencing steroidogenesis, cell metabolism, and growth through certain G protein-coupled receptors [6]. The two subunits that make up human FSH are the hormone-specific β subunit and the common α subunit found in all glycoprotein hormones. Both subunits go through significant post-translational modifications. FSHβ is encoded by the *FSHB* gene located on chromosome 11p21 [7].

The spermatogenetic process occurs within testicular seminiferous tubules, requiring several biochemical stimuli that are fine-controlled by both FSH and LH. Normal FSH concentrations are usually related to adequate spermatogonial abundance. On the other hand, endogenous FSH levels rise in situations when spermatogonia are absent or significantly reduced.

Through its receptor on the Sertoli cells, FSH gives spermatogenesis indirect structural and metabolic assistance, regulating the structural genes necessary for the metabolism and transfer of regulatory and nutritional chemicals from Sertoli to germ cells, as well as the genes involved in the structure of cell–cell junctions [8]. Moreover, it regulates the number of Sertoli cells, which is essential for spermatogenesis, controlling Sertoli cells’ mitotic proliferation, promoting their growth and maturation, and triggering the release of androgen-binding protein [9].

In addition, through its receptor, LH induces the secretion of testosterone in Leydig cells. In the testes, the concentration of testosterone is 50–100 times higher than in the peripheral circulation, and this pivotal aspect underlines that the environment inside the testes is crucial for maintaining and supporting spermatogenetic activity [10].

In summary, the primary role of FSH is to boost the number of sperm cells in synergy with intratesticular testosterone. An adequate FSH concentration, albeit not necessary for humans to complete spermatogenesis, is of paramount importance for sperm production. Previous in vivo studies demonstrated the effect of FSH (including different FSH preparations) on Sertoli cell activity [8,11].

When the first gonadotropic substance was isolated from human pituitary glands in the early 1960s, FSH was first used as infertility therapy. Many biosimilar and recombinant FSH molecules are currently available for purchase, in addition to extremely pure urinary FSH solutions [12]. The effects of recombinant and purified FSH (biosimilars and originators) appear to be similar, being well tolerated and safe [13]. FSH is often used in assisted reproduction techniques to induce multi-follicular growth in women, while its efficacy in treating men is not yet well established. In men, FSH therapy is used in patients with hypogonadotropic hypogonadism, in which this treatment is well known and established, alone or in combination with human chorionic gonadotropin (hCG) [3,14,15]. Indeed, the administration of exogenous hCG, alone or combined with FSH, restores spermatogenesis to varying degrees in up to 90% of patients with hypogonadotropic hypogonadism; as a result, spontaneous or assisted pregnancy rates have been recorded as high as 65% [9]. A similar technique is empirically advocated in male idiopathic infertility [16], and FSH therapy has been used to increase sperm quantity, quality, and pregnancy rate in patients with altered semen analysis parameters and FSH plasma levels below 8 IU/L, without signs of obstruction of the seminal tract [3].

Since there are FSH receptors in extra-gonadal tissues, it was suggested that FSH effects may extend beyond male fertility. However, it is widely recognized that FSH has no significant side effects, as confirmed by reports of patients with FSH-secreting pituitary adenoma, presenting testicular enlargement and no other systemic symptoms [17]. Furthermore, recent data point to the possibility that FSH may have extra-gonadal effects, such as those on bone metabolism, in light of the evidence that it binds to receptors on osteoclasts and appears to induce bone resorption. Moreover, poorer metabolic and cardiovascular outcomes have been linked to long-term exposure to elevated FSH levels, which may influence the cardiovascular system. Because FSHRs are expressed on immune cells and may have an impact on the inflammatory response, FSH has also been linked to the modulation of immune response [18]. Therefore, although FSH treatment is widely considered a safe treatment for MFI, larger RCTs are required to look at any possible extra-gonadal side effects of the FSH therapy course.

Furthermore, some concerns have been expressed about the cost of FSH treatment in idiopathic male infertility [19]. In this context, it has to be mentioned that in some countries (i.e., in Italy), FSH treatment is reimbursed by the National Health Service, without costs for the patient.

Regarding its effectiveness, previous data from 21 clinical trials demonstrated that when men with idiopathic infertility are given FSH [20], their sperm count and motility increase in a dose-dependent manner, a fact also highlighted by a meta-analysis of RCTs conducted by Cannarella et al. [21]. Moreover, previous clinical trials comprehensively suggest that FSH administration in idiopathic infertile men significantly increases pregnancy rate, both spontaneously and after assisted reproduction. However, the number of idiopathic infertile men who need to be treated to have one more pregnancy is estimated to be between 10 and 18 [22]. These results suggest that an increased success rate could be obtained by changing our approach to FSH administration, moving toward an overstimulating strategy. Similarly, the application of FSH in assisted reproduction has changed significantly over time. It has shifted from a fixed starting dose in the early years to a personalized approach in which FSH dosages are adjusted and increased according to the expected ovarian response. In clinical practice, FSH injections are frequently used in women to achieve the most oocytes feasible for assisted reproduction by inducing ovarian overstimulation [23]. Therefore, there are no endocrinological reasons why an FSH-mediated supraphysiological stimulus that is shown to be effective in increasing gamete production in the female gonad should not also be effective in male patients. However, to switch from a replacement strategy to an overstimulating strategy in the male scenario, it is imperative to determine pharmacologic markers of FSH efficacy. Furthermore, it should be useful in clinical practice to distinguish, before starting the treatment, among patients who might respond to FSH treatment. Indeed, although previous studies demonstrate the efficacy of FSH treatment in patients with idiopathic infertility [23], conflicting results are present in the literature, underlining that some populations of patients may not respond to FSH treatment [24] and that these populations of “non-responders” may account for about 50% of men who have normal levels of gonadotropin plasma. For this reason, identifying predictive markers of FSH action in spermatogenesis and clinical markers of response to FSH treatment is a fascinating area of study that may lead to new developments in personalized medicine for the treatment of male infertility.

More recently, Esteves SC et al. attempted, for the first time, to develop a system of criteria, named the APHRODITE criteria, based on clinical patient descriptions and the results of routine laboratory tests, including semen analysis and hormonal testing [25]. Five patient groups were delineated: (1) hypogonadotropic hypogonadism (acquired and congenital); (2) idiopathic male infertility with lowered semen analysis parameters, normal serum FSH, and normal serum total testosterone concentrations; (3) a hypogonadal state with lowered semen analysis parameters, normal FSH, and reduced total testosterone concentrations; (4) lowered semen analysis parameters, elevated FSH concentrations, and reduced or normal total testosterone concentrations; and (5) unexplained male infertility in the context of unexplained couple infertility. According to this classification, patients in group 1 may benefit from FSH and hCG treatment, patients in group 2 and 5 from FSH alone, while patients in group 3 might benefit from both FSH and hCG, although the response to FSH therapy in this group might mostly depend on the genetic background of the affected men [26]. No usefulness of FSH treatment is expected for patients in group 4. These criteria represent the first attempt of a patient stratification system of infertile male patients, aimed to improve reproductive outcomes following hormonal therapy [25]. Furthermore, other parameters, including sperm DNA fragmentation, testicular cytologic analysis, genetic markers, and post-genomic markers, have been called into question and might be useful to be integrated into a putative new clinical algorithm in order to predict the response to FSH treatment. This narrative review’s objective is to discuss the role of the different putative factors which have been proposed to predict the response to FSH treatment in idiopathic infertile men.

Previous studies, moreover, reported that higher doses of FSH [27], different injection intervals [28], or a longer treatment duration [29] may be associated with a higher degree of rescue of spermatogenesis in patients with idiopathic infertility.

## 2. Predictive Parameters of FSH Action

### 2.1. Biochemical Parameters

According to the most recent guidelines on the management of male infertility, FSH is suggested in selected patients with FSH concentrations below 8 IU/L [3]. Although the level of evidence is low, sufficient data support this potential therapeutic option [9,15]. On the other hand, only a few RCTs have been conducted on this topic of interest. Recent meta-analyses have verified the effectiveness of FSH in treating this particular group of patients with MFI [21,22,30,31]. In particular, these four meta-analyses revealed that greater pregnancy rates, less sperm DNA fragmentation, and enhanced sperm concentrations were linked to FSH treatment in male patients with idiopathic infertility. According to the available data, the only biochemical parameters which might foresee the response to FSH therapy is an FSH basal concentration below 8 IU/L. So far, no studies have suggested the potential efficacy of this hormonal empirical treatment in men with idiopathic infertility and basal FSH levels higher than 8 IU/L.

### 2.2. Semen Parameters

With the aim to identify seminal parameters able to predict FSH efficacy, a real-world study was recently conducted by Santi et al. [20], enrolling males who are idiopathically infertile and receiving 150 IU of FSH three times a week. Patients were treated until pregnancy achievement or for a maximum of two years. During treatment, two visits were considered: at baseline (V0) and after FSH treatment (V1). The authors reported that a V1-V0 percentage of sperm concentration higher than 30.8% predicted pregnancy. Furthermore, men with sperm concentrations <7.3 million/mL at baseline had a greater pregnancy rate. Therefore, sperm concentration at admission below 7.3 million/mL, thus increasing by more than 30.8% after treatment, might represent a marker for successful FSH treatment. This is the first, and still the sole, demonstration that semen parameters before and after FSH administration could be useful to predict its efficacy.

It was first proposed in 1998 that sperm DNA integrity is positively influenced by FSH [32]. In 2011, Palomba et al. [33] firstly reported that 150 IU of FSH administered on alternate days for 3 months was effective in reducing sperm DNA fragmentation (sDF). In this study, sperm chromatin dispersion was evaluated by the use of a specific kit (SPERM- HALOMAX KIT; INDAS Biotech, Madrid, Spain) in a qualitative way, classifying sperm chromatin dispersion into five patterns: sperm cells with large halos; sperm cells with medium-sized halos; sperm cells with very small-sized halos; sperm cells without a halo; and sperm cells without a halo-degraded. Since this first demonstration, several other studies confirmed this sperm quality improvement after FSH administration through the use of a terminal deoxynucleotidyl transferase-mediated deoxyuridine triphosphate (dUTP) in situ DNA nick end-labeling (TUNEL) assay [29,34,35,36,37]. In 2017, Garolla et al. [37] studied 166 infertile male candidates for assisted reproductive techniques. For three months, 84 individuals (cases) received FSH medication; 82 patients (controls) declined the treatment. After FSH treatment, patients’ seminal parameters and sDF indices significantly improved, while controls showed no changes at all. A total of 35 individuals (a pregnancy rate of 23.2%) were able to undergo intrauterine insemination thanks to FSH therapy. In total, 49 patients from the cases and all controls underwent intracytoplasmic sperm injection; the corresponding pregnancy rates were 23.2% and 40.8%. Treated patients obtaining a pregnancy had fewer double-strand breaks and a lower sDF score after three months of FSH medication, thus demonstrating that FSH treatment improves sDF and that double-strand breaks may represent a putative parameter in patients treated with FSH to predict reproductive outcome. More recently, Santi et al. [38] performed a meta-analysis to analyze the impact of FSH treatment on sDF, comprehensively confirming the beneficial FSH effect in patients with sDF > 20% independently of the sDF method applied, with an overall decrease in sDF of about 4.24% following therapy, indicating that this assay may be a useful one for assessing treatment response clinically. Thus, although several studies confirmed the potential role of FSH in improving the sperm DNA fragmentation index, no studies so far have been able to define its predictive role.

The predictive spermatic parameters for FSH response have been summarized in Table 1.

### 2.3. Testicular Fine-Needle Aspiration Cytological (FNAC) Analysis

First proposed in 1992, testicular fine-needle aspiration cytological (FNAC) analysis is a minimally invasive office-based technique that, with the use of a fine needle (usually 23 Gauge), permits the retrieval of testicular material from both testes. It has been suggested as a substitute for conventional biopsy in the assessment of males who are severely oligozoospermic and azoospermic [39]. Moreover, this evaluation might be useful in order to distinguish between obstructive and non-obstructive forms [3]. Testicular FNAC analysis permits the classification of spermatogenic alteration in Sertoli cell-only syndrome (SCOS), hypospermatogenesis, and germ cell maturation arrest. In fact, there may be further implications from determining the precise testicular change. Indeed, the identification of the specific testicular alteration might have further important implications for the treatment of patients with MFI.

The quantitative analysis of the cytological exam involves the quantification of the so-called spermatic index (the number of spermatozoa on the total number of spermatogenic cells) and the sertolian index (the number of Sertoli cells on the total number of spermatogenic cells).

**Figure 1:**
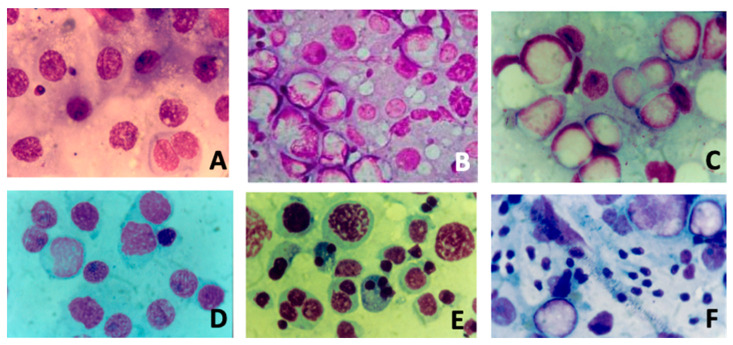
Different cytological pictures (Magnification 10×) from fine-needle aspiration of testis: (**A**) severe hypospermatogenesis; (**B**) moderate hypospermatogenesis; (**C**) maturative spermatogonial arrest; (**D**) maturative spermatocytic arrest; (**E**) maturative spermatidic arrest; (**F**) normal spermatogenesis with male tract obstruction.

Figure 1 summarizes the different cytological pictures from testicular FNAC analysis.

In the literature, several studies suggested that FSH treatment should be better proposed when testicular FNAC analysis highlights hypospermatogenesis without maturation arrest [3,13,16,40]. In particular, this was suggested by three studies from our group. Foresta et al. conducted a placebo-controlled, double-blind, randomized clinical study [41]. The authors assessed the tubular status and semen parameters of ninety oligozoospermic subjects both before and after FSH treatment. They discovered that the pretreatment testicular cytology results were consistent with hypospermatogenesis, which is linked to maturational disturbances at the spermatid level, in patients who did not respond to FSH treatment. Patients who responded to FSH treatment, on the other hand, had isolated hypospermatogenesis free of maturational abnormalities. A further randomized study [42] analyzed semen parameters and testicular cytology in 45 idiopathic oligozoospermic subjects with FSH plasma levels in the normal range, underlining that recombinant human FSH is effective in increasing the spermatogonial population and in promoting sperm production when a cytological picture of hypospermatogenesis without maturation arrest is present. Lastly, Garolla et al. [35] examined the prediction ability of testicular FNAC analysis and spermatid count in semen after FSH treatment. The authors found a strong correlation between hypospermatogenesis with maturative disruption and greater spermatid counts. In fact, in patients with lower spermatid counts, FSH treatment significantly improved sperm parameters and either natural or assisted fertility, indicating that spermatid count may be a potential predictor of responsiveness to FSH therapy.

Despite this evidence, data regarding testicular FNAC analysis as a diagnostic procedure in patients with MFI are limited, and this procedure is available in only a few centers, probably because of its invasive nature. However, beyond its diagnostic and therapeutic uses (such as in cases of obstructive azoospermia), it might be predictive of the outcome of FSH treatment and of subsequent sperm retrieval using testicular sperm extraction (TESE).

### 2.4. Genetics

The evaluation of FSH administration efficacy in male idiopathic infertility should be adjusted for confounding variables, such as pharmacogenetic markers, as ignoring them could produce unclear results. To our knowledge, there are currently only a few studies in which patients were selected and treated according to an FSH-based pharmacogenetic approach [43].

The effectiveness of FSH medication may be influenced by variations in the polymorphisms of the FSHB beta-subunit (FSHB) and FSH receptor (FSHR) genes [44]. The rate at which FSH is produced is limited by the transcription of FSHB, which regulates the amount of FSH secreted [45]. *FSHB* c.-211G > T polymorphism (rs10835638) is located within the promoter of the gene. According to functional tests, the wild-type promoter variant bearing the G-allele has twice the activity compared to FSHB c.-211G > T T homozygous. In particular, serum FSH levels are lower in the heterozygotes (GTs) and homozygotes (TTs) for the alternative allele than in the wild-type homozygotes (FSHB c.-211G > T G homozygous) [46]. Presumably, T-allele-induced disruption in the binding of the transcription factor LHX3 is the cause of the gene’s decreased transcriptional activity [45]. Thus, *FSHB* c.-211G > T T homozygous is linked to lower sperm counts, lower testicular volumes, and lower serum FSH levels [47]. Therefore, individuals with the T-allele do not sufficiently increase serum levels of FSH to achieve spermatogenesis. Previous studies, in particular one study from our group [48], suggested that infertile patients with *FSHB* c.-211G > T T homozygous better respond to FSH treatment, demonstrating, in comparison to carriers of the other genotype, a noteworthy increase in spermatogenesis following therapy. Actually, FSHB c.-211G > T homozygotes show a more pronounced rise in semen parameters, and TT homozygotes are much more likely than GT heterozygotes and GG homozygotes to become normozoospermic following therapy. In light of this evidence, in our routine clinical practice, *FSHB* c.-211G > T polymorphism analysis is used to identify a subgroup of infertile men who will respond well to FSH therapy and who have spermatogenic impairment and low or inappropriately normal FSH plasma levels. Nevertheless, conflicting data regarding this polymorphism exist in the literature. In particular, a recent study evaluating 1075 men undergoing TESE showed that *FSHB* c.-211G > T T homozygous was substantially linked to a lower likelihood of retrieving sperm [49]. In addition, *FSHB* c.-211G > T G homozygous was more frequently detected in patients with altered semen analysis in 190 infertile men, compared to 50 fertile controls [50]. Moreover, it was reported that *FSHB* c.-211G > T T homozygous was linked to a smaller testis size, but no significant correlation with other semen parameters—aside from a reduced number of morphologically normal spermatozoa in the heterozygous carriers—was found [51].

Another relevant polymorphism has been evaluated in the association of male infertility. *FSHR* c. 2039A > G p.N680S (rs6166) is well known to influence testicular volume in men [52], suggesting its potential role as a predictive marker of FSH treatment efficacy. Selice et al. [53] reported that sperm parameters improved after treatment only in patients with *FSHR* p.N680S S homozygous or heterozygous. On the contrary, Simoni et al. [26] found that *FSHR* p.N680S N homozygosity was associated with a decrease in the sperm DNA fragmentation index after FSH treatment, indicating that FSHR 326 p.N680S N homozygous responded to treatment. Thus, it is still unclear if pharmacogenetic approaches to FSH treatment are clinically useful due to inconsistent outcomes and a lack of evidence. Reasonably, the pharmacogenomic response to FSH administration should account for the combined effect of several *FSHB* and *FSHR* polymorphisms [43].

It is important to underline that the study of these polymorphisms related to FSH and its receptor is also underway in women, with the aim of understanding their cumulative impact on ovarian stimulation [54].

### 2.5. Post-Genomic Markers

Despite having its roots in genomic medicine, precision medicine has advanced significantly in order to unravel the intricacy of cellular physiology, and the current period is referred to as the “post-genomic era” [55]. Therefore, transcriptomics and proteomics might represent interesting platforms to identify putative markers of FSH efficacy in the management of male idiopathic infertility. It has been shown that the final cell response to FSH is determined by the intricate cooperation of a complex microRNA network and a complicated FSH signaling network. In vitro rat Sertoli cells have been shown to respond to FSH treatment through the expression of 163 microRNAs. The mentioned study identified the PTEN mRNA as one of the miRNA potential targets, whose 3′ region contains probable miR-23b and miR-217 target sites. In the vicinity of mature spermatids, FSH activity results in PTEN mRNA stability and protein accumulation in the apical region of the cells, potentially regulating cell adhesion [56]. Additionally, it has been suggested that miR-20a targets the VEGF mRNA, which is stimulated by FSH during spermiogenesis [57]. In addition, a study has demonstrated in rats the existence of microRNA targeting components, including miR-30c and miR-30, of the ERK pathway that are influenced by FSH action [58]. However, the study has not been followed by other clinical studies in humans.

Furthermore, proteomics has been used in in vitro models to attempt to identify new putative Sertoli markers of FSH action. Mancuso et al. demonstrated in porcine pre-puberal Sertoli cells that FSH stimulation induces an increase in inhibin-alpha, inhibin-beta, plakoglobin, haptoglobin, d-3-phosphoglycerate dehydrogenase, and sodium/potassium-transporting ATPase in extracellular vesicles, as reported in Table 2, thus representing putative markers of FSH action on Sertoli cells [8].

A further proteomic study was performed to compare the effect on porcine pre-pubertal Sertoli cells using several FSH preparations (α-follitropin, β-follitropin, and urofollitropin) in conjunction with testosterone [11], suggesting that the different preparations induce different effects in terms of specific proteomic markers, thus offering the chance to shed light on new applications for individualized reproductive medicine. Thus, promising results are available in the literature, although these parameters could still not be used in clinical practice.

## 3. Conclusions

FSH therapy induces significant semen improvements only in a proportion of patients with MFI. It is therefore advised to perform a complete and meticulous diagnostic workup of MFI to obtain presumptive or predictive data regarding the response to FSH treatment. Several studies, as presented here, have been included in few meta-analyses and the data often have several limitations, such as the empirical unstandardized use of FSH treatment, the heterogeneity of the patients with MFI enrolled, the heterogeneity of the studies included in the meta-analysis, and the different lengths of treatment with FSH.

Several studies have been produced and analyzed in the present review in order to understand which markers are prognostic of a good response to FSH treatment in male infertility, such as spermatid count, testicular cytology, and the assessment of the polymorphisms in both FSHR and FSHB genes. Moreover, further studies are being performed in order to identify new markers by using post-genomic platforms, including the identification of miRNAs and proteins, which need to be translated from bench side to clinical practice.

It is therefore time, as for FSH treatment for ovulation induction in women, to move from standard protocols toward an overstimulating personalized strategy. From this perspective, further studies aiming to detect actual markers of FSH administration efficacy are needed to create a tailored FSH therapy plan. The personalization of FSH treatment, based on the above discussed putative markers of response, is therefore mandatory to minimize side effects, to avoid lost time with ineffective treatments, and to improve the efficacy, predicting the most efficient dose and the duration of the treatment with FSH.

## Figures and Tables

**Table 1:** Predictive seminal parameters and cut-offs for FSH response.

Parameter	Cut-Off	Predictive for	References
V1–V0 percentage of sperm concentration	>30.8%	spontaneous pregnancy	[20]
sperm concentration	<7.3 million/mL	spontaneous pregnancy	[20]
sDF	>20%	improvement in sDF	[38]

**Table 2:** Putative proteomic markers of FSH action on Sertoli cells.

Proteins
Inhibin-alpha
Inhibin-beta
Plakoglobin
Haptoglobin
D-2-phosphoglycerate dehydrogenase
Sodium/potassium-transporting ATP-ase

## Data Availability

No new data were created or analyzed in this study. Data sharing is not applicable to this article.
